# Dispatcher-Assisted CPR in Italy: A Nationwide Survey of Current Practices and Future Challenges in Emergency Medical Communication Centers

**DOI:** 10.3390/jcm14020637

**Published:** 2025-01-19

**Authors:** Guglielmo Imbriaco, Giacomo Sebastiano Canova, Lorenzo Righi, Sara Tararan, Giorgia Di Mario, Nicola Ramacciati

**Affiliations:** 1 Department of Biomedicine and Prevention, University of Rome “Tor Vergata”, Via Montpellier 1, 00166 Rome, Italy; 2 118 Emilia Est Emergency Medical Communication Center, Maggiore Hospital, Largo Niglisoli 2, 40133 Bologna, Italy; 3 Scientific Committe, Italian Resuscitation Council, Via della Croce Coperta 11, 40128 Bologna, Italy; 4 118 Vicenza Emergency Medical Communication Center, AULSS 8 Berica, Vicenza, Viale F. Rodolfi, 37, 36100 Vicenza, Italy; giacomosebastiano.canova@aulss8.veneto.it; 5 Centrale Operativa 118 Siena-Grosseto, USL Toscana Sud Est, Strada del Ruffolo 2/A, 53100 Siena, Italy; lorenzo.righi@unisi.it; 6 Piattaforma Emergenza Urgenza, Azienda Sanitaria Friuli Occidentale, Via della Vecchia Ceramica 1, 33179 Pordenone, Italy; 7 Azienda Regionale Emergenza Sanitaria 118, Via Portuense 240, 00149 Rome, Italy; giorgiadm@live.it; 8 Department of Pharmacy, Health and Nutritional Sciences, University of Calabria, Via Alberto Savinio 54B, 87036 Rende, Italy

**Keywords:** cardiac arrest, cardiopulmonary resuscitation, dispatcher-assisted CPR, emergency medical communication center, out-of-hospital cardiac arrest, telephone CPR, survey

## Abstract

**Background/Objectives**: Dispatcher-assisted cardiopulmonary resuscitation (DA-CPR) is widely recognized as a critical intervention that significantly reduces no-flow time, improving survival rates in out-of-hospital cardiac arrests (OHCAs). This study evaluates current practices and the organization of DA-CPR in Italian emergency medical communication centers (EMCCs) and identifies areas for improvement. **Methods:** A cross-sectional survey was conducted between April and May 2024 among all Italian EMCCs, achieving a 92.6% response rate (62 out of 67) and covering 95.5% of the population. Data were collected on the availability of DA-CPR, additional medical instructions provided, standardized protocols, integration into dispatch software, availability of video call systems, and follow-up programs. **Results:** All responding EMCCs provide DA-CPR, with 79.1% (n = 49) initiating these protocols more than five years ago. In adult cardiac arrest, 74.2% (n = 46) provide instructions for chest compressions only. Standardized protocols are used in 69.4% (n = 43) of EMCCs, and 53.2% (n = 33) have these protocols integrated into their dispatch software. Additionally, 93.5% (n = 58) provide dispatcher-assisted instructions for other medical conditions, including pediatric CPR (90.3%, n = 56), neonatal CPR (90.3%, n = 56), foreign body airway obstruction (85.5%, n = 53), labor (56.5%, n = 35), and massive bleeding (41.9%, n = 26). A training path for DA-CPR is available in 48 EMCCs (77.4%), and in most cases, it is included in the basic dispatcher course (56.5%, n = 36), with 50% conducting periodic retraining. Moreover, 33.9% (n = 21) utilize video call systems to support dispatcher-assisted instructions. Data on DA-CPR are collected by 46.8% of EMCCs (n = 29), primarily on relevant cases, but only 25.8% (n = 16) have a follow-up path for patients. **Conclusions:** This study highlights a widespread implementation of DA-CPR across Italian EMCCs. However, regional disparities, mainly in protocols and technological support, indicate areas requiring urgent attention. Enhancing training programs and standardizing protocols could improve DA-CPR effectiveness and patient outcomes, thus guaranteeing equitable care nationwide. Future initiatives should focus on integrating support tools like video calls, expanding retraining programs, and establishing follow-up and debriefing paths.

## 1. Introduction

Immediate cardiopulmonary resuscitation (CPR) on the scene significantly improves overall outcomes of out-of-hospital cardiac arrest (OHCA) patients, increasing survival rates by two to three times [1]. Despite its critical importance, training laypeople in CPR requires time, resources, and periodic retraining, and may be difficult to realize in a broader context.

Emergency medical communication center (EMCC) providers represent a critical interface in the first phases of the chain of survival, playing an essential role in strengthening the collaborative effort between the public and emergency medical services (EMSs) [2,3]. Dispatcher-assisted CPR (DA-CPR) has been shown to effectively enhance the rate of bystander-initiated resuscitation and to significantly improve patient outcomes during OHCAs [4,5].

The role of DA-CPR in improving OHCA survival was included in the European Resuscitation Council (ERC) guidelines in 2010 [6], emphasized with a strongly recommended intervention in 2015 [7]. Afterward, DA-CPR practices were implemented worldwide, with different strategies and organizations. International studies conducted in Europe, Australia and New Zealand, and the United States (USA) reported that the percentage of EMCCs providing DA-CPR varied from 51% to 100% [8,9,10,11]. In 2016 and 2019, the Italian Resuscitation Council promoted two surveys to investigate the provision of DA-CPR in Italy, reporting a heterogeneous and suboptimal organization [12,13].

Given the importance of dispatcher-assisted instructions (DA-Is) in reducing the therapy-free interval and consequently optimizing outcomes, a specific survey was performed. The aim of this research was to evaluate the current organization of Italian EMCCs on this particular topic, providing a comprehensive assessment of current practices and variabilities and identifying critical areas for improvement in the Italian context.

## 2. Materials and Methods

### 2.1. Study Design

A descriptive observational cross-sectional survey was performed between April and May 2024, involving all Italian EMCCs (n = 67, 27 in Northern Italy, 15 in Central Italy, and 25 in Southern Italy and islands). This paper is structured according to the Consensus-Based Checklist for Reporting of Survey Studies (CROSS) (Supplementary file S1) [14].

### 2.2. Study Setting

Health care services in Italy are administered on a regional basis, following national recommendations on essential levels of assistance. This has led to differentiated organization and performance across the country. The current organization of emergency medical services in Italy started in 1992, following an act of coordination issued by the Government, leading to the implementation of the number 1-1-8 as the national number for medical emergencies. The implementation of the European emergency number 1-1-2 as a centralized first-level public service answering point (PSAP) for all emergency services (police, fire, and medical) is in progress.

Emergency medical dispatch centers in Italy have a diverse territorial organization, with some EMCCs covering individual provinces while others manage a broader territory, including several provinces or an entire region. EMCCs are staffed by trained dispatchers, nurses, or physicians, who assess the urgency of calls and guarantee the dispatch of emergency response units using local or regional protocols. To improve cardiac arrest response, the provision of DA-CPR instructions by EMCCs has recently been included in national law (2021) [15].

### 2.3. Data Collection Methods

A specific questionnaire was developed to fulfill the research aim. The survey tool was developed following international experiences reported in the literature. Ten nurses with experience in emergency medical dispatch were involved in the validation process. The content validity of individual questions (I-CVI) ranged from 0.83 to 1, and the overall questionnaire validity (S-CVI) was 0.96. The content validity evaluation showed that all the items were rated as relevant by the expert panel, reaching acceptable I-CVI and S-CVI/Ave values according to literature recommendations [16]. The face validation of the questions in the final version of the questionnaire obtained an average score of 3.71 (±0.53) on a scale from 1 to 4. Questionnaire development, validation, and pre-testing were detailed in a previous publication [17].

The tool is divided into six sections: general information, dispatcher-assisted cardiopulmonary resuscitation, the availability of dispatcher-assisted instructions for clinical conditions other than adult cardiac arrest, education and retraining for dispatcher-assisted instructions, the integration of new technologies, the availability of follow-up, and audit processes. The total number of questions is 38 (the full version of the questionnaire is available as Supplementary file S2).

### 2.4. Sample Characteristics

**Figure 1:**
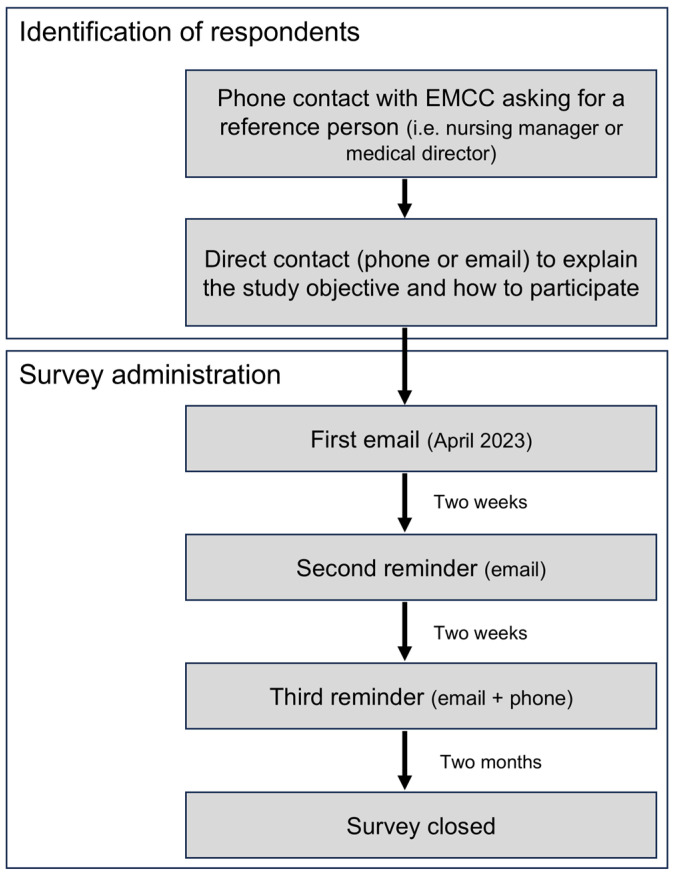
Process of identification of the respondents (one single reference person for every emergency medical dispatch center—EMCC) and survey administration.

The survey protocol was designed to obtain one single answer from every dispatch center. Accordingly, the research group identified a reference person contacting all Italian EMCCs by phone and asking for the direct contact of a nursing manager or a medical director. Subsequently, the reference person was contacted by email or phone to present the project, asking for participation and clearly explaining that the questionnaire had to be completed by one single person for every EMCC. The process of the identification of the respondents and the survey administration are depicted in Figure 1.

### 2.5. Survey Administration

The questionnaire was sent to the EMCC contact person at the beginning of April 2024. The online survey, developed with Forms, Office 365 (Microsoft, Redmond, VA, USA), was shared via email. In the case of no response, a second reminder email was sent after two weeks. Following another two weeks, non-responders were contacted by phone and email by a researcher. Failure to complete the survey following the third reminder was considered a no-response. The data collection was closed after two months (Figure 1).

### 2.6. Ethical Considerations

This study was submitted for review to the local Ethical Committee (CE-AVEC), which determined that formal approval was not required as the survey does not qualify as clinical research involving health data. The project was endorsed by the Italian Resuscitation Council.

Survey participants were assured of confidentiality throughout the data collection process. Personal identifiers (name, role, and email address) were collected with the only aim of checking the effective participation of the EMCC. All data were stored in a secure, password-protected database, accessible only to the research team. Additionally, any identifying information was removed three months after closing the survey, and results are reported in aggregate form.

### 2.7. Statistical Analysis

Data were analyzed using basic descriptive statistics, including frequencies, percentages, and means, computed using Excel, Office 365 (Microsoft, Redmond, VA, USA).

## 3. Results

**Figure 2:**
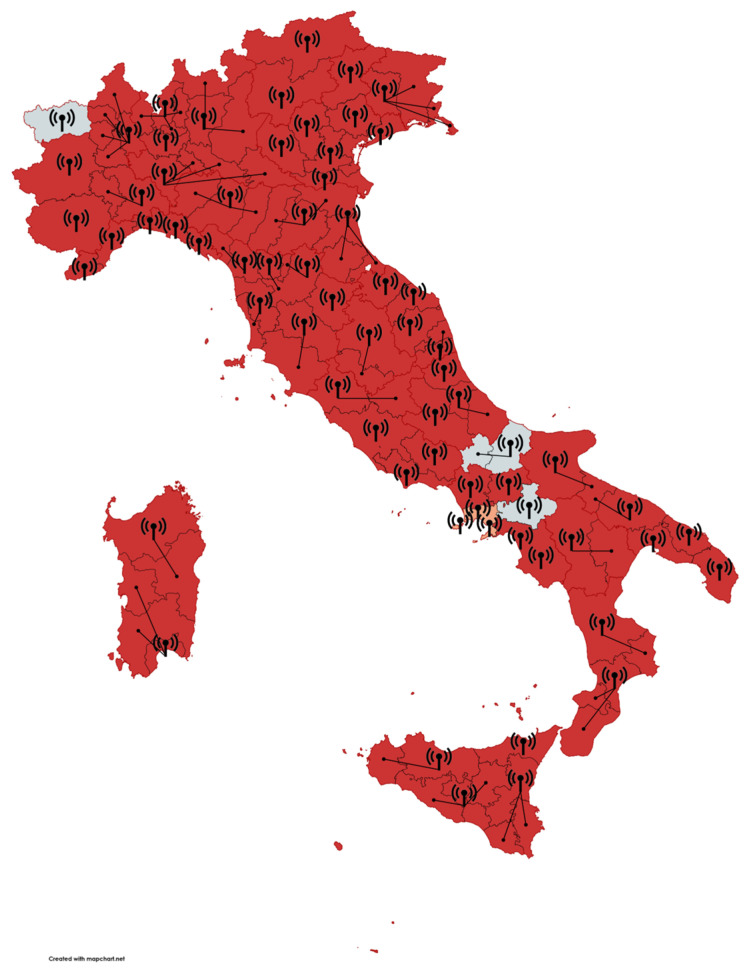
Map of Italian Emergency Medical Communication Centers (EMCCs) participating in the survey. The antenna icons show the location of EMCCs and the provinces served (connected with lines). In the province colored in light red, one EMCC out of three answered the questionnaire. White EMCCs did not participate in the survey. Map created with Mapchart, www.mapchart.net–accessed on 30 November 2024.

The survey achieved a response rate of 92.6% (62/67 EMCCs), covering 95.5% of the Italian population. Regional participation rates varied: Northern Italy (96.3%, 26/27), Central Italy (100%, 15/15), and Southern Italy and islands (84%, 21/25) (Figure 2).

A total of 30 EMCCs (44.8%) responded to the first invitation. An additional 14 (20.9%) answered following a second email. Finally, after a further reminder, 18 more centers (26.9%) completed the survey. The questionnaire was answered by 47 nursing managers (75.8%), 10 medical directors (16.1%), and 5 specialized nurses (8.1%). No multiple participations were registered.

The 1-1-2 European emergency number is currently operational in 71% (n = 44) of the surveyed EMCCs.

The composition of the staff includes different occupational categories. Technical operators are present in 56.5% (n = 35) of EMCCs, with an average presence of 16.5% (SD 21.7); in 6 EMCCs, their presence is above 50%. Nurses are present in all dispatch centers, with an average of 69.9% (SD 23.4): in 83.9% (n = 52), the presence is higher than 50%, and in 58.1% (n = 36), it is higher than 75%; 6 EMCCs employ only nursing staff. Physicians are present in 80.6% (n = 50), with an average of 12.9% (SD 10.4).

### 3.1. Main Findings

#### 3.1.1. Dispatcher-Assisted Cardiopulmonary Resuscitation

All responding EMCCs provide DA-CPR, with 74.2% (n = 46) using chest-compression-only protocols in cases of adult cardiac arrest. In most cases, rescue breaths are proposed in the case of trained or collaborative bystanders, a cardiac arrest of respiratory etiology, an absence of risk or contraindications, and the availability of devices (i.e., a pocket mask). In the case of pediatric cardiac arrest, 72.6% of EMCCs (n = 45) provide instructions for standard CPR (chest compressions and rescue breaths).

DA-CPR instructions are based on a standardized protocol in 69.4% (n = 43) of EMCCs. DA-CPR scripts are included in the EMCC software (56.5%, n = 35), available in other electronic formats (e.g., PDF files) (46.8%, n = 29), or other forms like action cards or printed sheets (43.5%, n = 27). Detailed information is reported in Table 1.

In 90.3% of EMCCs (n = 56) CPR instructions are managed by the first dispatcher who answers the emergency call; in 5 EMCCs, a cardiac arrest call is transferred to another provider with a dedicated role or higher qualification.

#### 3.1.2. Availability of Dispatcher-Assisted Instructions for Other Clinical Conditions

**Figure 3:**
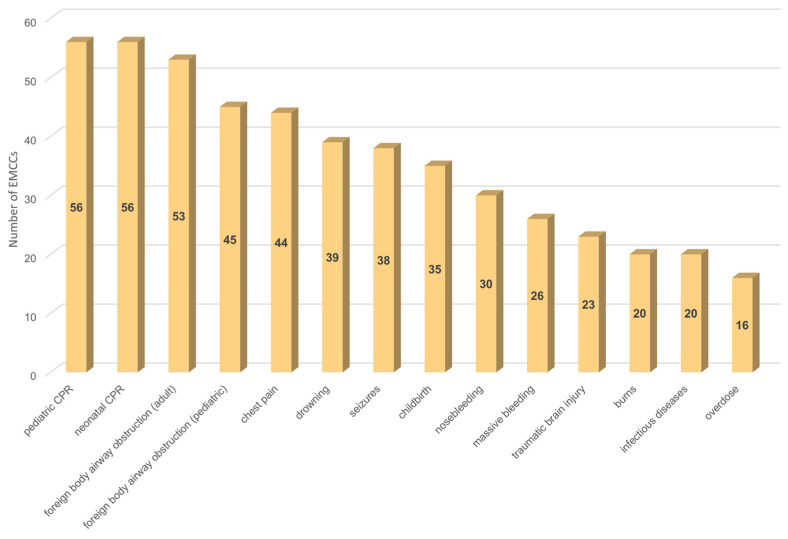
Availability of dispatcher-assisted instructions for other clinical conditions. Abbreviations: CPR, cardiopulmonary resuscitation; EMCCs, emergency medical communication centers.

Fifty-eight (93.5%) Italian EMCCs have pre-arrival instructions for other medical conditions, in addition to cardiac arrest (Figure 3). The most widespread are pediatric and neonatal CPR (both 90.3%, n = 56), followed by instructions for foreign body airway obstruction for adults (85.5%, n = 53) and pediatrics (72.6%, n = 45).

#### 3.1.3. Education and Retraining for Dispatcher-Assisted Instructions

Training on DA-CPR is available in 77.4% (n = 48) of EMCCs, with most including it in the basic dispatcher course (70.8%, n = 34). The educational approaches are various and feature a combination of classroom teaching (67.7%, n = 42), listening to real emergency calls (56.5%, n = 35), simulations (41.9%, n = 26), supervised call taking on real calls (46.8%, n = 29), and role-playing (37.1%, n = 23). A retraining course is available in half of the surveyed Italian EMCCs (n = 31) (Table 1).

#### 3.1.4. Integration of New Technologies and Support Systems

The availability of video call systems, instant text messaging, and digital maps or registries of automated external defibrillators (AEDs) is detailed in Table 2. Only three centers reported the full implementation of apps to alert nearby first responders in cardiac arrest cases. The other 16 EMCCs reported the possibility of alerting additional resources in the case of cardiac arrest nearby, such as the police or firefighters, or the contact person responsible for a defibrillator, in most cases via a phone call. This information is not fully captured by this survey and requires a specific investigation. Notably, the availability of these support systems is significantly different between Northern, Central, and Southern Italy.

#### 3.1.5. Follow-Up and Audit Processes

Data collection on DA-CPR is performed by 46.8% (n = 29) of EMCCs, focusing primarily on relevant cases. Follow-up on patient outcomes is available in 25.8% (n = 16) of centers. Structured audit processes are reported by 74.2% (n = 46) of EMCCs, in most cases for particularly relevant events (n = 17) or upon dispatchers’ request (n = 14). A specific reporting system, for example, in the case of bystander refusal to perform DA-CPR, is available in 24 centers (38.7%). Debriefing paths for responders are available in 45.2% of EMCCs (n = 28).

## 4. Discussion

This study offers a comprehensive picture of the current state of DA-CPR practices across Italian EMCCs, with a high response rate covering over 95% of the Italian population. Despite significant geographical differences, similar international studies achieved lower response rates, ranging from 54% to 81.5% [8,9].

All participating EMCCs provide DA-CPR instructions, underscoring the country’s commitment to emergency cardiac care, as mandated by a 2021 law based on the ERC “Systems saving lives” guidelines [1,15]. The universal provision of DA-CPR indicates broad acceptance of this collaborative approach in OHCA cases, aligning with international best practices. Previous studies describe differentiated rates of DA-CPR: 100% in Australia and New Zealand and 27 out of 28 European countries [10,11]. Two surveys performed in the USA showed that EMCCs increased DA-CPR availability from 51% in 2010 to 91.9% in 2022 [8,9]. Additionally, over 70% of Italian EMCCs have been implementing DA-CPR for more than a decade, demonstrating a long-established experience. However, despite this widespread availability, our findings reveal substantial regional disparities, variations in protocol availability, and limitations in technology integration that could impact OHCA outcomes.

The regional variability in DA-CPR response and protocol adherence among Italian EMCCs is consistent with findings from previous surveys [12,13]. The Northern and Central areas show higher adherence to standardized DA-CPR practices, while Southern regions exhibit greater variability. Such regional differences align with previous studies investigating pain management options in the Italian prehospital emergency setting [18,19], suggesting the need for a deeper comprehension of the reasons behind the regional differences in the implementation of EMS. Notably, international observations reported similar challenges across Europe and ILCOR member countries [11,20]. Standardizing DA-CPR protocols [21] and expanding training across all regions may help bridge these gaps, ensuring consistent and high-quality CPR instructions throughout the whole country.

The availability of 1-1-2 public service answering points (PSAPs) in a little more than two-thirds of cases reflects partial but growing integration of this European-standard emergency response system across the country.

The staffing composition in Italian EMCCs reflects a multidisciplinary approach that could enhance the quality of DA-CPR; on the other hand, dispatcher expertise might vary, potentially influencing OHCA response. This approach is similar to Australia or New Zealand, where emergency calls are not routinely answered by a paramedic or other medically trained personnel and can be transferred to a clinician [10]. Contrarily, in countries like the USA, dispatch roles are more standardized, often filled by certified providers (i.e., dispatchers or telecommunicators) trained specifically for emergency communication [9]. The authors believe that employing nurses in EMCCs represents an added value because of their better knowledge of clinical variations in patient status. The Italian approach could be strengthened by introducing additional training requirements for all staff involved in DA-CPR, ensuring consistent skill levels and reducing potential variability related to different qualifications.

While several EMCCs in Italy have adopted innovative tools like video calls and AED mapping, these technologies are not universally available. Only three EMCCs can dispatch first responders via a dedicated app [22]. The limited availability of video support may hinder optimal OHCA responses, especially in Southern regions. Studies showed that technology-assisted DA-CPR, including video instruction, can enhance bystander confidence and CPR quality [23,24]. Similarly, AED registries are not consistently available in Europe, being present in 21 out of 28 countries [11]. Expanding the use of video calls and ensuring all EMCCs have access to AED location data and the additional dispatch of first responders could facilitate a more rapid and effective emergency response, potentially improving OHCA outcomes [25,26].

Our results reveal a notable gap in quality control and feedback systems within Italian EMCCs, with less than half maintaining DA-CPR data collection systems and even fewer (25.8%) implementing patient outcome follow-ups. Quality control, through structured audits and feedback loops, is vital for evaluating dispatcher performance and identifying areas for improvement. A series of performance measures for DA-CPR quality assessments have been proposed by the American Heart Association and include the rate of OHCA correctly identified and where DA-CPR was provided, and the times of OHCA recognition (goal < 90 s) and the beginning of chest compressions (goal <150 s) under a dispatcher’s guidance [27]. Despite the survey by Sutter et al. (2015) underscoring the importance of emergency call review and feedback mechanisms in enhancing DA-CPR quality and effectiveness [8], it is worth considering that performance metrics may be difficult to achieve in real-world settings and its assessment requires consistent resources [28]. Furthermore, disseminating data on patient outcomes with dispatchers improves awareness and performances on cardiac arrest identification and the provision of DA-CPR [29,30]. Implementing regular audits and structured debriefings may represent, for Italian EMCCs, an opportunity for continuous improvement in DA-CPR. Moreover, sharing positive experiences of DA-CPR and OHCA survival within the community may improve the collaborative interaction between EMCCs and the public [31].

This survey’s findings suggest several potential improvements at both organizational and policy levels. The regional variations reported in the present study call for a deeper exploration of the barriers to the implementation of DA-I practices to guarantee an equitable distribution of resources not only for EMCCs but for the whole Italian EMS organization.

Specific guidelines on dispatchers’ activities, based on international recommendations and experiences and contextualized to the Italian setting, represent a necessary step for the development of national DA-I protocols. Secondly, structured dispatcher training programs, associated with a broader implementation of retraining programs and simulations would ensure that dispatchers maintain high competency in DA-CPR. Scientific societies may contribute to the definition of a core curriculum for dispatchers and specific education programs. Additionally, expanding the adoption of video calls and AED mapping and standardizing technological resources and software already in use in some EMCCs could improve emergency response capability. Finally, considering a broader perspective of data collection, such as a national cardiac arrest registry, implementing systematic quality assurance and feedback mechanisms could support dispatcher development, thus encouraging continuous improvement.

### Strength and Limitations

This survey was specifically focused on DA-CPR practices and provided a comprehensive and updated overview of EMCC organizations in Italy. Previous studies investigating a broader data set on EMS organizations included only limited information on dispatchers’ activities. Despite the data collection being designed to carefully avoid duplicate compilations, using a validated questionnaire and asking for collaboration with a single contact person per EMCC, this study’s reliance on self-reported responses may introduce limitations due to potential response bias and regional variability in protocol reporting. Additionally, the authors acknowledge that first responders’ activation systems are a complex and multifaceted topic requiring a dedicated investigation.

## 5. Conclusions

This nationwide survey among Italian EMCCs provides a comprehensive overview of current DA-I practices, highlighting both progress and ongoing challenges in Italy’s DA-CPR framework. The findings indicate that DA-CPR is universally implemented, with 74.2% of EMCCs providing chest-compression-only instructions for adult cardiac arrests. However, regional disparities persist in protocol standardization, the integration of technological support tools such as video calls, and follow-up programs. Only 46.8% of EMCCs collect DA-CPR data, and 25.8% reported having a structured patient follow-up system. Training is available in roughly three-quarters of centers, yet periodic retraining remains inconsistent.

These results underscore the need for the development of national protocols, enhanced training programs, and a wider integration of support technologies to ensure the equitable provision of DA-Is across regions. By addressing these areas, Italian EMCCs may enhance the quality and effectiveness of their DA-CPR response, improving outcomes for OHCA patients. Future initiatives should involve scientific societies, professional bodies, and policymakers, specifically aimed at overcoming regional or local variabilities and standardizing emergency responses across regions.

## Figures and Tables

**Table 1:** Dispatcher-assisted CPR activity and protocols.

	North	Center	South and Islands	Italy
Number of respondent EMCCs	26	15	21	62
Experience in DA-CPR				
<1 year	-	-	1 (4.8)	1 (1.6)
1–2 years	-	-	1 (4.8)	1 (1.6)
2–5 years	2 (7.7)	3 (20.0)	6 (28.6)	11 (17.7)
5–10 years	2 (7.7)	1 (6.7)	2 (9.5)	5 (8.1)
>10 years	22 (84.6)	11 (73.3)	11 (52.4)	44 (71.0)
Chest-compression-only DA-CPR				
Adult	21 (80.8)	9 (60.0)	16 (76.2)	46 (74.2)
Pediatric	7 (26.9)	1 (6.7)	9 (42.9)	17 (27.4)
DA-CPR provision				
Fixed script/protocol	25 (96.2)	9 (60.0)	9 (42.2)	43 (69.4)
Main scheme	1 (3.8)	5 (33.3)	5 (2.8)	11 (17.7)
Freely managed by dispatchers	-	1 (6.7)	7 (33.3)	8 (12.9)
Integration of DA-CPR into the EMCC software				
Yes	20 (76.9)	8 (53.3)	5 (23.8)	33 (53.2)
No	6 (23.1)	6 (40.0)	14 (66.7)	26 (41.9)
N/A	-	1 (6.7)	2 (9.5)	3 (4.8)
Specific training in DA-CPR	23 (88.5)	10 (66.7)	15 (71.4%)	48 (77.4%)
Included in the basic dispatcher course	15 (57.7) *	9 (90.0) *	12 (80.0) *	34 (70.8) *
Dedicated course	3 (11.5) *	1 (10.0) *	3 (20.0) *	7 (14.6) *
Certified course by external agencies	5 (19.2) *	-	-	5 (10.4) *
Retraining courses for DA-CPR				
>1 per year	2 (7.7)	1 (6.7)	1(4.8)	4 (6.5)
1 year	2 (7.7)	1 (6.7)	-	9 (14.5)
1 every 2 years	8 (30.8)	1 (6.7)	5 (23.8)	8 (12.9)
Not on a regular schedule/undefined	5 (19.2)	2 (13.3)	3 (14.3)	10 (16.1)
Not available	9 (34.6)	10 (66.7)	12 (57.1)	31 (50.0)

Data reported as numbers and percentages: N (%). * Percentages refer to the number of EMCCs providing specific training in dispatcher-assisted CPR. Abbreviations: DA-CPR, dispatcher-assisted cardiopulmonary resuscitation; EMCC, emergency medical communication center; N/A, data not available/not provided.

**Table 2:** Availability of new technologies and support systems for dispatcher-assisted instructions.

	North	Center	South and Islands	Italy
Number of respondent EMCCs	26	25	21	62
Video calls Text messaging	12 (46.2) 9 (34.6)	8 (53.3) 5 (33.3)	1 (4.8) 14.3% (3)	21 (33.9) 17 (27.4)
AED maps	26 (100)	13 (86.7)	12 (57.1)	51 (82.3)
First responders Activated with a dedicated app Activated via phone call Other/unclear *	10 (38.5) 3 (11.5) 7 (26.9) -	2 (13.3) - 2 (13.3) -	7 (33.3) - 2 (9.5) 5 (23.8)	19 (30.6) 3 (4.8) 11 (17.7) 5 (8.1)

Data reported as numbers and percentages: N (%). * Participating EMCCs did not provide detailed information on their first responder activation systems. Abbreviations: AED, automated external defibrillator; EMCCs, emergency medical communication centers.

## Data Availability

The data presented in this study are available upon request from the corresponding author.

## Supplementary Materials

The following supporting information can be downloaded at https://www.mdpi.com/article/10.3390/jcm14020637/s1: File S1: Checklist for reporting of survey studies (CROSS); File S2: questionnaire.
