# Supplementary material for: Dispatcher-Assisted CPR in Italy: A Nationwide Survey of Current Practices and Future Challenges in Emergency Medical Communication Centers
Source: J Clin Med. 2025 Jan 19;14(2):637. doi: 10.3390/jcm14020637 (PMC11766162; doi:10.3390/jcm14020637)
Supplement: Supplementary file 1 [file jcm-14-00637-s001.zip › S2 - Questionnaire.pdf]

Dispatcher-Assisted CPR in Italy: A Nationwide Survey of Current Practices and Future Challenges in Emergency Medical Communication Centers

Supplement 2 - Questionnaire

**Section 1 – General information**		
Q1a	Name of the emergency medical communication service	(free text answer)
Q1b	City (location of the Emergency Medical Dispatch Center)	(free text answer)
Q1C	Availability of the European Emergency Number 1-1-2	• Yes<br>• No
Q2	Area served (name/s of the cities/towns/provinces served by the emergency medical communication center)	(free text answer)
Q3	Population served (approximately)	(free text answer)
Q4	Professional qualifications of providers, and respective percentages of each group (e.g. technicians 80%, nurses 15%, doctors 5%)"	• Technicians<br>• Nurses<br>• Physicians
Q5a	Name/surname	(free text answer)
Q5b	Professional role within the emergency medical communication center	(free text answer)
Q5c	Email address	(free text answer)
**Section 2 – Dispatcher-assisted CPR**		
Q6	Does your service provide Dispatcher-Assisted CPR in case of cardiac arrest?	• Yes<br>• Yes, only for adult patients<br>• No
Q7	How many years have you been providing Dispatcher-Assisted CPR in case of cardiac arrest in your emergency medical communication center?	• <1 year<br>• 1-2 years<br>• 2-5 years<br>• 5-10 years<br>• 10 years<br>• Don't know<br>• Not applicable
Q8a	What are the criteria used by your center to recognize cardiac arrest and begin the provision of Dispatcher-Assisted CPR?<br>(please, indicate all the answers that apply)	• Unconscious + not breathing<br>• Unconscious + not breathing normally<br>• Unconscious + breathing uncertain/unknown<br>• Other

		• Not applicable
Q8b	Does your dispatch center employ advanced technologies such as AI, speech recognition, or video analytics during emergency calls?	• Yes — please specify<br>• No<br>• Don't know<br>• Other
Q9a -	What are the components of Dispatcher-Assisted CPR instructions for cardiac arrest in adult patients at your center, in case of untrained bystanders?	• Chest compressions only<br>• Chest compressions and rescue breaths<br>• Other<br>• Not applicable
Q9b	What are the components of Dispatcher-Assisted CPR instructions for pediatric cardiac arrest at your center, in case of untrained bystanders?	• Chest compressions only<br>• Chest compressions and rescue breaths<br>• Other<br>• Not applicable
Q10	Dispatcher-Assisted CPR instructions are given:	• Strictly according to a fixed script<br>• Dispatchers use their own words following a main scheme<br>• Freely managed<br>• Other<br>• Not applicable
Q11	In which formats are Dispatcher-Assisted CPR instructions available to dispatchers? (please, indicate all the answers that apply)	• Electronic format integrated into the dispatch center software<br>• Other electronic formats (e.g. PDF files)<br>• Printed sheets<br>• Action cards or similar<br>• Other<br>• Nothing<br>• Not applicable
Q12	Are Dispatcher-Assisted CPR instructions integrated into the Emergency Medical Dispatch Center software (e.g. MDPS)?	• Yes<br>• No<br>• Our dispatch center does not use any computer software<br>• Not applicable
Q13	Are CPR instructions provided by the first dispatcher handling the emergency call or is the call transferred to another provider with higher qualification? (e.g. nurse or	• Provided by the first provider handling the call

	physician)	• Transferred to another provider with specific/dedicated role (e.g. supervisor)<br>• Transferred to another provider with higher qualification<br>• Other<br>• Not applicable
**Section 3 - Dispatcher-assisted instructions for other clinical conditions**		
Q14	Does your emergency medical communication center have any other dispatcher-assisted instruction available for clinical conditions other than cardiac arrest in adult patients?	• Yes<br>• No<br>• Not applicable
Q14b	If YES, which other Dispatcher-assisted instructions are provided by your dispatch center?<br>(please, indicate all the answers that apply)	• Pediatric cardiac arrest<br>• Neonatal cardiac arrest<br>• Chocking/foreign body airway obstruction (adult)<br>• Chocking/foreign body airway obstruction (pediatric)<br>• Chest pain<br>• Drowning<br>• Seizures<br>• Labor<br>• Nosebleed<br>• Massive bleeding/hemorrhage control<br>• Traumatic brain injury<br>• Burns<br>• Infectious diseases<br>• Overdose<br>• Other<br>• Don't know<br>• Not applicable
**Section 4 - Training and education**		
Q15a	Is the provision of Dispatcher-assisted instructions included in an educational/training program?	• Yes<br>• No
Q15b	If yes, is the training program for Dispatcher-assisted instructions:	• Included in the basic dispatcher course<br>• In a specific training path<br>• A certified course by external agencies

		• Other<br>• Not applicable
Q15c	Which educational strategies are used to teach dispatcher-assisted interventions? (please, indicate all the answers that apply)	• Classroom teaching/lessons<br>• Role-playing<br>• Simulation of emergency calls<br>• Listening to real calls<br>• Supervised call-taking<br>• Other<br>• Not applicable
Q16a	Does your service organize retraining courses on dispatcher-assisted CPR?	• Yes<br>• No
Q16b	If yes, at what interval?	• >3 times/year<br>• 2 times/year<br>• 1 times/year<br>• < 1 time/year<br>• Undefined/not on a regular schedule<br>• Don't know/other<br>• Not applicable
**Section 5 – New technologies**		
Q17a	Which communication tools are used in your dispatch center to provide dispatcher-assisted instructions?<br>(please, indicate all the answers that apply)	• Phone<br>• Video<br>• Text messages<br>• Other<br>• Don't know<br>• Not applicable
Q17b	If your dispatch center uses video calls, which systems/instruments are used?	• (free text answer)
Q18a	In case of suspect or confirmed cardiac arrest, do you have a system of first-responder (e.g. police, firefighters, volunteers, lay rescuers) activation?	• Yes<br>• No
Q18b	If yes, which categories of first responders are alerted/activated?<br>(please, indicate all the answers that apply)	• CPR-trained citizens<br>• Untrained citizens<br>• Volunteers (including organized volunteer groups)<br>• Police<br>• Firefighters<br>• Other<br>• Don't know<br>• Not applicable

Q19	Do you have a mapping system of Automated External Defibrillators available in your territory?	• Yes<br>• No
Q19b	If your Emergency Medical Dispatch Center has an AED map, which devices are available?<br>(please, indicate all the answers that apply)	• public/city-owned AEDs<br>• private AEDs<br>• Schools<br>• police<br>• firefighters<br>• volunteer associations<br>• other<br>• don't know<br>• Not applicable
**Section 6 - Data collection and follow-up programs**		
Q20a	Does your service have a system to collect data on Dispatcher-assisted instructions?	• Yes<br>• No<br>• Not applicable
Q20b	If YES, on which types of Dispatcher-assisted instructions the data is collected?	• Always<br>• Only for cardiac arrests<br>• Only for relevant cases<br>• Other<br>• Don't know<br>• Not applicable
Q21a	Is there a structured audit process for cases where Dispatcher-assisted instructions have been provided?	• Yes, always<br>• Yes, only on relevant cases<br>• Yes, on random emergency calls<br>• Yes, upon dispatcher's request<br>• Other<br>• No<br>• Don't know
Q21b	Does your service have a feedback form for dispatchers (e.g. in case of refusal of dispatcher's instructions)	• Yes<br>• No<br>• Not applicable
Q22	Does your service have a structured follow-up/debriefing path (e.g. survival rate, neurological outcome) for patients where Dispatcher-assisted CPR has been provided?	• Yes<br>• Yes, only for cardiac arrests<br>• Yes, only for relevant cases<br>• No<br>• Other<br>• Not applicable

Q23	Is there a follow-up/debriefing path for bystanders who have performed dispatcher-assisted CPR?	• Yes<br>• Yes, only for cardiac arrests<br>• Yes, only for relevant cases<br>• No<br>• Other<br>• Not applicable
